# Positive perception and efficacy of compression stockings for prevention of lower limb edema in pregnant women

**DOI:** 10.1590/1677-5449.210101

**Published:** 2022-01-31

**Authors:** Orlando Adas Saliba-Júnior, Hamilton Almeida Rollo, Orlando Saliba, Marcone Lima Sobreira

**Affiliations:** 1 Universidade Estadual Paulista “Júlio de Mesquita Filho” – UNESP, Botucatu, SP, Brasil.

**Keywords:** varicose veins, edema, stockings, compression, pregnant women, controlled clinical trial

## Abstract

**Background:**

Pregnancy is characterized by physiological changes that can contribute to development of varicose veins, venous insufficiency, and leg edema.

**Objectives:**

To evaluate the effect of compression stocking on lower limb edema in pregnant women and their perceptions of wearing them.

**Methods:**

This was a randomized, controlled, prospective, parallel, blinded clinical trial conducted with 60 pregnant women randomly distributed into two groups: an intervention group (n = 30) wearing compression stockings and a control group (n = 30). Standardized ankle and calf measurements were taken of all 120 lower limbs using a tape measure. At the end of the study, a questionnaire was administered to identify perceived difficulties and advantages related to wearing compression stockings.

**Results:**

Pregnant women in the intervention group had a significantly smaller increase (p < 0.05) in calf and ankle diameters compared to those in the control group. The mean differences from the beginning to the end of gestation in the diameters of the right calf, left calf, right ankle, and left ankle respectively were 0.30 cm, 0.30 cm, 0.15, cm and 0.15 cm in the intervention group and 1.95 cm, 1.95 cm, 1.73 cm, and 1.87 cm in the control group. Most of the pregnant women had no difficulty wearing the compression stockings and all reported that they felt a difference in leg symptoms and would wear stockings again.

**Conclusions:**

Compression stockings were effective for preventing lower limb edema in pregnant women, who had a positive perception of wearing them.

## INTRODUCTION

In women, pregnancy is one of the factors that most contributes to the prevalence of varicose veins, which in turn can trigger venous insufficiency and leg edema.^1^ The prevalence of varicose veins during pregnancy varies greatly and, in addition to regional and racial differences, this is due to use of different concepts and classifications and also to the type of epidemiological analysis performed. Estimates vary from 20 to 50% of pregnant women and when all types of varicose veins are included, such as telangiectasias for example, the prevalence can climb as high as 70%.^2^ The physiological changes that take place during pregnancy can be an important contributing factor to the increased incidence of varicose veins, venous insufficiency, and leg edema in women.^3^ The most common symptoms of varicose veins are edema and pain, in addition to nocturnal cramps, dormancy, tingling, and feelings of heaviness in the lower limbs. Treatments for varicose veins are generally divided into three main groups: surgery, pharmacological treatments, and non-pharmacological treatments. Treatments for leg edema primarily consist of reduction of symptoms using pharmacological and non-pharmacological approaches.^3^^,^ 4

Compression therapy can be used for conditions that involve lower-limb venous and lymphatic insufficiency, as is the case with varicose veins. The several forms of compression therapy include elastic and inelastic bandages, boots, stockings, and pneumatic devices.^5^^-^ 7 Compression is also recommended for inflammatory conditions that include an edema component, which can include cellulitis, some forms of vasculitis, and other medical treatments.^4^

Compression stockings constitute a noninvasive treatment option for venous and lymphatic diseases.^4^ Evidence shows that this therapeutic approach can even relieve the symptoms of varicose veins and edema in people whose professions require them to spend long periods standing upright, such as hairdressers.^8^

A recent randomized clinical trial demonstrated the efficacy of compression stockings for controlling varicose veins in the lower limbs of pregnant women by measuring the diameters of the great saphenous vein (GSM) and small saphenous vein with duplex ultrasonography and assessing clinical symptoms of pain, edema, and feelings of heaviness in the lower limbs.^9^

One advantage of compression stockings is that they are relatively simple to use compared to pneumatic devices and bandages.^10^ However, few studies have assessed multiparous pregnant women’s perceptions of the advantages, disadvantages, and difficulties related to wearing compression stockings.^11^^,^ 12 Another important point that should be acknowledged is the need to assess the perceptions of women in different countries, considering differences that may exist in terms of behavioral and cultural aspects. The objectives of this study were to assess the effect of compression stockings on lower limb edema using standardized ankle and calf measurements and to investigate perceptions of wearing them in pregnant Brazilian women.

## METHODOLOGY

### Study type

This is a randomized, controlled, blind, prospective clinical trial conducted with pregnant women distributed at random into two groups. The study was registered on the Brazilian Register of Clinical trials (REBEC, RBR-2HP7RS) under number UTN U1111-1175-7723.

### Study population

The study population comprised pregnant women who sought care at the Gynecology and Obstetrics Department of a public university hospital.

The sample size was calculated using the *z* test for two proportions, assuming an unknown result, 95% reliability (5% significance level), 5% margin of error, and 80% test power. The minimum sample size was 27 pregnant women in each group; however, this was increased by approximately 10% to account for possible losses, resulting in 30 pregnant women in each group.

The study enrolled healthy, white-skinned, pregnant women at 10 to 15 weeks’ of a uterine pregnancy, aged 18 to 40 years, with normal distal pulses (pedal or posterior tibial), who signed a free and informed consent form agreeing to take part in the study after being informed about the study objectives and procedures.

Women were excluded if they exhibited any of the following conditions during the initial examination: complaints or clinical evidence of arterial, lymphatic, or orthopedic disease; excess weight greater than 10% of body mass index; any known degenerative disease in course; limb edema not of venous origin; deep venous thrombosis (DVT); prior DVT confirmed by an objective examination (duplex ultrasonography); clinical, etiology, anatomic, and pathophysiologic classification (CEAP) of 4, 5, or 6 because of skin changes that could interfere with wearing of compression stockings and/or make it difficult or impossible to take the measurements; and reflux time greater than 1.0 seconds in deep vein system. It was necessary to approach 110 registered pregnant women to obtain the sample size needed (n = 60).

Small cards were made bearing the numbers 01 to 60 and placed into sealed opaque envelopes. Each volunteer was given an envelope at random. Those whose envelopes contained cards 01 to 30 comprised the intervention group and those who had cards numbered 31 to 60 comprised the control group.

In the intervention group, 6.67% of the pregnant women had a CEAP classification of 0; 13.33% had CEAP = 1; 40.00% had CEAP = 2; and 40.00% had CEAP = 3. None of the women in the control group had a CEAP classification of 0; 50.00% had CEAP = 1; 33.33% had CEAP = 2; and 16.67% had CEAP = 3. At the start of their gestations, 73.33% of the pregnant women in the intervention group reported painful lower limbs; 50.00% reported lower limb edema; and 66.67% reported feelings of heaviness in the lower limbs. Among the pregnant women in the control group, 33.33% reported pain in the lower limbs; 16.67% reported lower limb edema; and 33.33% reported feelings of heaviness in the lower limbs. None of the pregnant women in either of the groups reported signs of redness at the start of the study.

The members of the intervention group wore 20-30 mmHg knee-length compression stockings (BASIC model, Sigvaris® brand) for approximately 8 h per day from the first clinical and ultrasonographic examination, at the start of the study, until the second assessment, at the end of the gestation. During this period, they were monitored every fortnight by telephone to check on regular use of the compression stockings.

Each intervention group participant was given three pairs of stockings, sized to fit on the basis of the following dimensions: diameters of ankle and calf and length of the leg from the calcaneus to the knee. When they were given the stockings, the women were also given verbal and written instructions on how to wear them and told that if they needed replacements because of damage caused by the long period of use they should contact the team immediately to be given new stockings.

Standardized ankle and calf measurements were taken of all 120 lower limbs with a tape measure. Ankle diameter was measured 3 cm above the medial malleolus and calf diameter was measured 10 cm below the tuberosity of the tibia (at the largest diameter). The diameter measurement procedure was conducted with the patient standing upright, during the afternoon, between 16:00 and 17:00, and compression stockings were removed 1 h before the examination. The initial and final examinations were conducted, respectively, between the 10th and 13th weeks and between the 30th and 33rd weeks of gestation. The researchers responsible for performing these examinations were unaware of group membership and did not have access to clinical charts or interview notes. The volunteers were instructed not to talk about wearing compression stockings with the professionals responsible for performing the examinations.

Data were collected on the age, height, and weight of the women studied and noted on a dedicated form. At the end of the study, a questionnaire was administered during interviews with the pregnant women, asking about perceived difficulties and advantages related to wearing compression stockings.

### Statistical analysis

A descriptive analysis of the data was conducted and statistical tests were applied after the normality of data distributions had been verified. Comparisons were made between initial and final data and between intervention and control groups. The *t* test for paired samples was used to compare initial and final data and the *t* test for independent samples was used to compare groups. Tests were performed using GraphPad InStat 3.0 ® and Bioestat v. 5.3 software.^13^ A 5% significance level was adopted in all tests.

### Ethical considerations

The study was approved by the Research Ethics Committee (protocol 4362- 2012) and was conducted in accordance with National Health Council (Conselho Nacional de Saúde) resolution 466/2012. Authorization was also requested and obtained from the Teaching Health Center to recruit pregnant women to participate in the study. The study complies with all ethical principles required for this type of study, as set out in the Helsinki Declaration and the Nuremberg Code. A free and informed consent form was drawn up and all of the women were given clear and objective information about the study.

## RESULTS

The mean age of the pregnant women studied was 27.03 years in the intervention group and 26.07 years in the control group. At the start of the study, mean weight and height were 67.29±9.46 kg and 1.64 m respectively in the intervention group and 63.73±11.31 kg and 1.65 m respectively in the control group. At the end of the study, mean weight was 77.96±10.75 kg in the intervention group and 75.57±12.00 kg in the control group. There were no significant differences (p < 0.05) in the physical characteristics of the two groups at the start or the end of the study.

Table 1 shows the results for calf and ankle diameter measurements at the start and end of gestation. It was observed that the pregnant women in the intervention group exhibited smaller increases in the diameters of both calf and ankle than those in the control group.

**Table 1:** Results of measurements of calf and ankle diameters (cm) of pregnant women at the start and end of pregnancy. Botucatu, SP, Brazil, 2017.

**Group**	**Site**	**Leg**	**Examination**	**Mean**	**Median**	**Standard deviation**	**Minimum**	**Maximum**	***p*-value**
Intervention	Calf	Right	Initial	37.40	37.25	3.15	30.00	43.00	0.0174
			Final	37.70	37.50	3.37	29.50	43.50	
		Left	Initial	37.62	37.75	3.41	31.00	44.00	0.0174
			Final	37.92	38.00	3.51	30.00	44.00	
	Ankle	Right	Initial	22.97	23.00	1.85	19.00	26.00	0.1635
			Final	23.12	23.00	2.10	18.00	26.00	
		Left	Initial	23.08	23.00	1.90	19.00	26.00	0.1527
			Final	23.23	23.00	2.10	18.50	27.00	
Control	Calf	Right	Initial	35.62	35.00	3.73	30.00	44.00	< 0.0001
			Final	37.72	37.00	3.82	32.00	45.00	
		Left	Initial	35.69	35.00	3.86	30.00	45.00	< 0.0001
			Final	38.02	37.00	4.10	32.50	46.00	
	Ankle	Right	Initial	21.75	22.00	1.96	18.00	25.00	< 0.0001
			Final	23.62	24.00	2.21	19.50	28.50	
		Left	Initial	21.92	22.00	1.91	18.50	25.00	< 0.0001
			Final	23.90	24.00	2.17	20.00	28.50	

The mean differences, from start to end of gestation, for the diameters of the right calf, left calf, right ankle, and left ankle were, respectively, 0.30 cm, 0.30 cm, 0.15 cm, and 0.15 cm in the intervention group and 1.95 cm, 1.95 cm, 1.73 cm, and 1.87 cm in the control group (Table 2).

**Table 2:** Mean differences between calf and ankle diameters (cm) of pregnant women, measured at the start and end of pregnancy. Botucatu, SP, Brazil, 2017.

**Site**	**Leg**	**Group**				***p*-value**
		**Intervention**		**Control**		
		**Mean**	**Standard deviation**	**Mean**	**Standard deviation**	
Calf	Right	0.30	0.65	1.95	0.99	< 0.0001
	Left	0.30	0.65	1.95	1.14	< 0.0001
Ankle	Right	0.15	0.57	1.73	0.73	< 0.0001
	Left	0.15	0.56	1.87	0.96	< 0.0001

Table 3 lists the data on the pregnant women’s perceptions of wearing compression stockings. The majority of the pregnant women stated they did not have any problems with putting on or wearing the compression stockings. All of the pregnant women stated that they felt a difference in leg symptoms and would wear stockings again.

**Table 3:** Distribution of pregnant women according to their perceptions of wearing compression stockings. Botucatu, SP, Brazil, 2017.

**Questions about wearing compression stockings**		**Yes**		**No**	**Total**	
	**n**	**%**	**n**	**%**	**n**	**%**
Did you have any difficulties with wearing compression stockings?	11	36.67	19	63.33	30	100.00
Did you need help to put the compression stockings on?	6	20.00	24	80.00	30	100.00
Did you feel any difference in leg symptoms related to wearing compression stockings?	30	100.00	0	0.00	30	100.00
When you were wearing them, did you ever have to take the compression stockings off?	11	36.67	19	63.33	30	100.00
Would you wear compression stockings again?	30	100.00	0	0.00	30	100.00

## DISCUSSION

In this study about use of compression stockings by pregnant women, it was observed that the increases in calf and ankle diameters were smaller among pregnant women who wore the compression stockings compared with those in the control group, and that the measure was well tolerated by the patients.

Varicose veins can be defined as subcutaneous veins that have undergone dilation, tortuosity, or stretching that are clinically palpable or visible when the patient is standing upright.^14^^,^ 15 They can cause undesirable clinical manifestations, including edema, pain, congestion, skin irritation, muscle cramps, weight, tension, and feelings of swelling in the lower limbs.^15^ Non-pharmacological interventions for treatment of leg edema and varicose veins include wearing elastic compression stockings, raising the leg, all forms of rest, exercise, reflexology, immersion in water, physiotherapy, and massage.^3^^,^ 16

Lower limb edema is very common during pregnancy, affecting around 80% of all pregnant women, and primarily occurs during the third trimester of gestation, when it can be considered physiological edema.^17^^,^ 18 Several different techniques can be used to assess the levels of edema in the lower limbs, including measuring the circumference of the leg, which can be accomplished with a tape measure or with a more elaborate form of tape measure, the Leg-O-Meter.^18^ Other methods, such as rheoplethysmography, extensometer plethysmography, and air plethysmography, can be used to assess changes related to lower limb edema.^18^ Other methods for measurement of lower limb edema include optoelectronic assessment, computed tomography, magnetic resonance, and dual-energy x-ray absorptiometry. However, these methods are expensive and difficult for the population to access, in comparison with the method used in the present study.^18^ In a recent study by Saliba et al.,^9^ it was found that 70% of pregnant women who did not wear stockings complained of edema. Corroborating the findings of the present study, these authors also observed that prevalence of pain, edema, and sensations of swelling in the lower limbs at the end of the gestation were all reduced in the group of pregnant women who had worn compression stockings, demonstrating the efficacy of this treatment.^9^

It can be conjectured that the effect in terms of prevention of increase in calf and ankle diameters could actually be even greater, since there is no way to guarantee that the compression stockings were not used incorrectly, although measures were taken to control the treatment. Monitoring of compression stocking use was conducted by fortnightly phone calls, which was extremely important for the study, since it proved necessary to replace stockings because of wear and also to reschedule days and times set for examinations.

The intervention group members’ perceptions of using compression stockings demonstrate the ease of use of this prophylactic and therapeutic measure. All of these pregnant women reported that they felt a difference in leg symptoms and that they would wear compression stockings again. The findings of this study are in agreement with a study about acceptance of compression stockings conducted by Allegra et al.,^19^ who found that leg symptoms and pain were reduced in pregnant women who wore compression stockings. These authors also observed that improvement of symptoms was associated with regularity of wearing stockings, demonstrating the importance of wearing them continuously to improving the quality of life of pregnant women.^19^

Important questions related to the efficacy of stockings include: the compression needed to achieve the desired effects and what effect the compression has on limb volume. Results of a study of wearing compression stockings, with a prescription of 20-30 mmHg compression for 8 h per day from the 12th week of gestation onwards, showed that there was reflux in the GSM and the small saphenous vein in 0/30 patients in the treatment group and in 16/30 in the group that did not wear the stockings, analyzed by Doppler ultrasonography examination at the end of the gestation.^20^ Thaler et al.^21^ employed stockings with differing levels of compression and found reflux at the saphenofemoral junction in 1/27 patients in the treatment group and 4/15 in the control group. A study conducted using computed tomography to observe in vivo the effect provoked by elastic stockings in superficial veins when lying down found that 36 mmHg compression reduced the caliber of the GSM by 70%.^22^ These findings provide evidence of the importance of the correct choice of protocol in terms of the prescription for wearing compression stockings in order to obtain the desired results.

Recognizing DVT and pulmonary embolism as among the most important causes of morbidity and mortality in pregnancy, a cohort study was conducted with pregnant women seen at a Pregnancy Health-care Program at an obstetric clinic run by a hospital in Cremona, Italy, to assess the effects for prevention of these complications of wearing 12-18 mmHg above-the-knee compression stockings, with and without concomitant administration of low molecular weight heparin. The authors concluded that both interventions were safe and useful for reducing the incidence of venous thromboembolism among pregnant women.^23^ Moreover, there is also evidence to suggest that wearing 20-30 mmHg compression stockings could be adopted as a preventative measure to minimize the risk of venous thromboembolism in pregnant women during long-distance travel.^24^

In this study, the pregnant women wore the stockings for at least 8 h per day. They were monitored by telephone and this aspect was covered in terms of periods of the day (morning, afternoon, and night). They were not asked to keep an exact record of the time spent wearing compression stockings, since to impose too many conditions could have reduced participation. It was notable that even though 1/3 of the pregnant women had faced some difficulty related to wearing the compression stockings and had had to take them off at some point during the study, no long periods elapsed without wearing them, since these situations were sporadic occurrences related to a need to replace stockings because of damage caused by constant use. In such cases, the women had been instructed to contact the researchers for immediate replacement, emphasizing the importance of continued adherence to treatment to achieving the desired results.^20^ The fact that the pregnant women were monitored via telephone may have been a positive factor in compliance with the recommendations for wearing compression stockings, and, to a certain extent, made it possible to identify the difficulties experienced. A study by Adamczyk et al.^25^ demonstrated that compression therapy was well-tolerated by pregnant women and that improvement in symptoms was perceived when they were worn daily for at least 4 h.

It should be noted that the present study enrolled both women in their first pregnancy and women who were in their second or subsequent pregnancy, and the groups were balanced in terms of this variable. This is a strong point of this study because the majority of studies in the scientific literature only recruited primiparous women. Studies demonstrate that multiparous women are at greater risk of developing varicose veins over time, irrespective of weight gain associated with pregnancy.^11^^,^ 26 These changes are present in approximately 13% of primiparous women, 30% of those in their second gestation, and up to 57% of multiparous women.^26^ There is also evidence that multiparity (≥ 4 deliveries) constitutes a relevant risk factor for occurrence of venous thromboembolism during pregnancy.^12^

It was concluded that compression stockings were an effective measure for prevention of lower-limb edema in pregnant women, who reported positive perceptions of wearing them.
