# Supplementary material for: Positive perception and efficacy of compression stockings for prevention of lower limb edema in pregnant women
Source: J Vasc Bras. 2022 Jan 31;21:e20210101. doi: 10.1590/1677-5449.210101 (PMC8958436; doi:10.1590/1677-5449.210101)
Supplement: Translation

# Translation: Percepção positiva e eficácia das meias de compressão na prevenção de edema em membros inferiores de gestantes

**Authors:** Orlando Adas Saliba-Júnior, Hamilton Almeida Rollo, Orlando Saliba, Marcone Lima Sobreira

**Affiliations:** 1 Universidade Estadual Paulista “Júlio de Mesquita Filho” – UNESP, Botucatu, SP, Brasil.

**Keywords:** varizes, edema, meias de compressão, gestantes, ensaio clínico controlado

## Abstract

**Contexto:**

A gravidez é caracterizada por mudanças fisiológicas que podem contribuir para o desenvolvimento de varizes, insuficiência venosa e edema das pernas.

**Objetivos:**

Avaliar o efeito das meias de compressão em edema de membros inferiores e a percepção sobre o uso por gestantes.

**Métodos:**

Trata-se de um ensaio clínico randomizado, controlado, prospectivo, paralelo e cego realizado com 60 mulheres grávidas distribuídas aleatoriamente em dois grupos: grupo intervenção (n = 30), que usou meias de compressão, e grupo controle (n = 30). Foram realizadas medições padronizadas de tornozelo e panturrilha, empregando-se fita métrica, em todos os 120 membros inferiores. No final do estudo, foi aplicado também um questionário para verificação das dificuldades e vantagens percebidas com relação ao uso das meias de compressão.

**Resultados:**

As gestantes do grupo intervenção apresentaram aumento significativamente menor (p < 0,05) nos diâmetros de panturrilha e tornozelo em relação ao grupo controle. As diferenças médias, no início e no final da gestação, nos diâmetros de panturrilha direita, panturrilha esquerda, tornozelo direito e tornozelo esquerdo foram de, respectivamente, 0,30 cm, 0,30 cm, 0,15 cm e 0,15 cm no grupo intervenção e 1,95 cm, 1,95 cm, 1,73 cm e 1,87 cm no grupo controle. A maioria das gestantes não teve dificuldade para utilizar as meias de compressão, e todas relataram que sentiram diferença nos sintomas das pernas e que usariam as meias novamente.

**Conclusões:**

As meias de compressão foram eficazes na prevenção de edema em membros inferiores de gestantes, as quais apresentaram percepção positiva quanto à sua utilização.

## INTRODUÇÃO

A gestação é um dos fatores que mais contribui para a prevalência de varizes em mulheres, podendo desencadear insuficiência venosa e edema na perna^1^. Durante a gestação, a prevalência de varizes varia bastante, e isso se deve, além das diferenças regionais e raciais, à utilização de diferentes conceitos e classificações, e até ao tipo de análise epidemiológica realizada. Essa estimativa varia de 20 a 50% das grávidas e, quando são incluídos todos os tipos de varizes, como, por exemplo, as telangiectasias, a prevalência pode elevar-se, chegando a 70%^2^. As mudanças fisiológicas que ocorrem durante a gravidez podem ser um fator importante que contribui para o aumento da incidência de varizes, insuficiência venosa e edema das pernas em mulheres^3^. Os sintomas mais comuns de varizes são edema e dor, além de cãibras noturnas, dormência, formigamento e sensação de peso nos membros inferiores. Os tratamentos para varizes são geralmente divididos em três grupos principais: cirurgia, tratamentos farmacológicos e não farmacológicos. Os tratamentos de edema de perna compreendem principalmente redução de sintomas por meio de abordagens farmacológicas e não farmacológicas^3^^,^ 4.

A terapia compressiva pode ser utilizada em condições que envolvem insuficiência venosa e linfática nos membros inferiores, tais como as varicosidades. As várias formas de terapia de compressão incluem bandagens elásticas e não elásticas, botas, meias e dispositivos pneumáticos^5^^-^ 7. A compressão também é recomendada para condições inflamatórias que possuem um componente de edema, as quais podem incluir celulite, algumas formas de vasculite e outros tratamentos médicos^4^.

As meias de compressão são consideradas uma opção de tratamento não invasivo para doenças venosas e linfáticas^4^. Evidências demonstram que essa medida terapêutica pode inclusive aliviar os sintomas de varizes e edema em indivíduos com profissões que exigem períodos prolongados em pé, tais como cabeleireiros^8^.

Recente estudo clínico randomizado demonstrou a eficácia das meias de compressão no controle das varizes em membros inferiores de gestantes por meio da avaliação dos diâmetros da veia safena magna (VSM) e da veia safena parva por ultrassonografia dúplex e dos sintomas clínicos de dor, edema e sensação de peso nos membros inferiores^9^.

As meias de compressão têm a vantagem de serem relativamente mais simples de utilizar em comparação aos dispositivos pneumáticos e de bandagem^10^. Entretanto, poucos estudos avaliaram a percepção de gestantes multíparas sobre as vantagens, desvantagens e dificuldades de uso das meias de compressão^11^^,^ 12. Outro aspecto importante a ser ressaltado refere-se à necessidade de se avaliar a percepção de mulheres de diferentes países, considerando as diferenças que porventura possam existir quanto aos aspectos comportamentais e culturais. O objetivo deste estudo foi avaliar o efeito das meias de compressão em edema de membros inferiores, por meio de medições padronizadas de tornozelo e panturrilha, e a percepção sobre o uso por gestantes brasileiras.

## METODOLOGIA

### Tipo de estudo

Trata-se de um ensaio clínico randomizado, controlado, prospectivo, paralelo e cego realizado com mulheres grávidas distribuídas aleatoriamente em dois grupos. O estudo foi registrado no Registro Brasileiro de Ensaios Clínicos (REBEC, RBR-2HP7RS) com o número UTN U1111-1175-7723.

### População estudada

A população de estudo foi composta por gestantes que demandaram atendimento no Serviço de Ginecologia e Obstetrícia do hospital de uma faculdade pública de medicina.

Para o cálculo amostral, foi empregado o teste *z* para duas proporções, supondo resultado desconhecido, com confiabilidade de 95% (nível de significância de 5%), margem de erro de 5% e poder do teste de 80%. O tamanho amostral mínimo foi de 27 gestantes para cada grupo; entretanto, foi ampliado em aproximadamente 10%, considerando as possíveis perdas, resultando em 30 gestantes para cada grupo.

Foram incluídas no estudo gestantes saudáveis, com 10 a 15 semanas de gravidez tópica, com idade entre 18 a 40 anos, de cor branca, com pulsos distais normais (pedioso ou tibial posterior) e que assinaram um termo de consentimento livre e esclarecido (TCLE) concordando em participar da pesquisa, após terem recebido informações sobre os objetivos e a realização da pesquisa.

As mulheres grávidas que apresentaram alguma das seguintes condições durante o exame inicial foram excluídas: queixas ou evidências clínicas de doença arterial, linfática ou ortopédica; excesso de peso acima de 10% do índice de massa corporal; qualquer doença degenerativa conhecida em andamento; edema de membros que não fosse de origem venosa; trombose venosa profunda (TVP); TVP prévia comprovada por exame objetivo (ultrassonografia dúplex); classificação clínica, etiológica, anatômica e patofisiológica (CEAP) 4, 5 ou 6 em função de alterações na pele que poderiam interferir na questão do uso das meias de compressão e também dificultar ou inviabilizar as medições propostas; e tempo de refluxo maior que 1,0 segundo no sistema venoso profundo. Foi necessária a abordagem de 110 gestantes cadastradas para obtenção do número final estabelecido (n = 60).

Foram confeccionados pequenos cartões com números de 01 a 60, os quais foram colocados em envelopes opacos lacrados. Cada gestante recebeu aleatoriamente um envelope. Aquelas que receberam números de 01 a 30 compuseram o grupo de intervenção, e as que receberam os números de 31 a 60 compuseram o grupo controle.

No grupo de intervenção, 6,67% das gestantes apresentavam classificação CEAP = 0; 13,33%, CEAP = 1; 40,00%, CEAP = 2; e 40,00%, CEAP = 3. No grupo controle, nenhuma das gestantes apresentava classificação CEAP = 0; 50,00%, CEAP = 1; 33,33%, CEAP = 2; e 16,67%, CEAP = 3. No início da gestação, verificou-se que, entre as gestantes do grupo de intervenção, 73,33% reportavam dor nos membros inferiores; 50,00%, edema nos membros inferiores; e 66,67%, sensação de peso nos membros inferiores. Entre as gestantes do grupo controle, por sua vez, 33,33% relatavam dor nos membros inferiores; 16,67%, edema nos membros inferiores; e 33,33%, sensação de peso nos membros inferiores. Nenhuma das gestantes de ambos os grupos relatou sinais de vermelhidão no início do estudo.

O grupo intervenção fez uso de meia, de comprimento até o joelho, com compressão de 20-30 mmHg, modelo BASIC da marca Sigvaris®, por aproximadamente 8 h diárias, desde o primeiro exame clínico e ultrassonográfico, no início do estudo, até a segunda avaliação, no final da gestação. Durante esse período, as gestantes foram monitoradas quinzenalmente, por telefone, quanto ao uso regular das meias de compressão.

Cada gestante recebeu três pares de meia, de tamanho adequado à perna, considerando-se as seguintes dimensões: diâmetros do tornozelo e da panturrilha e altura da perna (do calcâneo até joelho). Juntamente com as meias, as gestantes receberam orientações verbais e por escrito sobre o modo de uso, bem como a informação de que, se houvesse necessidade de reposição por motivos de danos causados ao longo do período de uso, elas deveriam entrar em contato imediato para recebimento de novas meias.

Foram realizadas medições padronizadas de tornozelo e panturrilha, empregando-se fita métrica, em todos os 120 membros inferiores. O diâmetro do tornozelo foi verificado 3 cm acima do maléolo medial, e o da panturrilha 10 cm abaixo da tuberosidade da tíbia (maior diâmetro). O procedimento de mensuração dos diâmetros foi realizado com a paciente em posição ortostática, no período da tarde, das 16 h às 17 h, e as meias de compressão eram retiradas 1 h antes do exame. Os exames inicial e final foram realizados, respectivamente, entre a 10ª e 13ª e entre a 30ª e 33ª semana de gestação. Os pesquisadores responsáveis pela realização dos exames não tinham conhecimento da composição dos grupos, bem como não tinham acesso às fichas clínicas e aos formulários das entrevistas. As gestantes foram orientadas a não comentar sobre o uso das meias de compressão com os profissionais responsáveis pela realização dos exames.

Os dados referentes à idade, altura e peso das mulheres estudadas foram obtidos e registrados em formulário específico. No final do estudo, foi aplicado também um questionário, em entrevista com as gestantes, para verificação das dificuldades e das vantagens percebidas com relação ao uso das meias de compressão.

### Análise estatística

A análise descritiva dos dados foi realizada e os testes estatísticos foram empregados após verificação da normalidade das distribuições de dados. Foram feitas comparações dos dados iniciais e finais, bem como comparações entre os grupos. O teste *t* pareado foi utilizado para comparação de dados iniciais e finais, e o teste *t* para amostras independentes para comparação entre os grupos. Para tanto, foram utilizados os softwares GraphPad InStat 3.0 ® e Bioestat v. 5.3^13^. Em todos os testes, foi adotado o nível de significância de 5%.

### Aspectos éticos

O estudo foi aprovado pelo Comitê de Ética em Pesquisa (protocolo 4362- 2012) e foi realizado de acordo com a resolução do Conselho Nacional de Saúde (CNS) 466/2012. Também foi solicitada e obtida a autorização do Centro de Saúde Escola para o recrutamento das gestantes para participação na pesquisa. Foram respeitados os preceitos éticos exigidos para esse tipo de estudo, estabelecidos na resolução citada, bem como na declaração de Helsinki e no Código de Nuremberg. Foi elaborado o TCLE, e todas as gestantes receberam informações claras sobre os objetivos e o desenvolvimento da pesquisa.

## RESULTADOS

A idade média das gestantes estudadas foi de 27,03 anos no grupo intervenção e 26,07 anos no grupo controle. No início do estudo, o peso e a estatura médios foram, respectivamente, 67,29±9,46 kg e 1,64 m para o grupo intervenção e 63,73±11,31 kg e 1,65 m para o grupo controle, Ao final do estudo, o peso médio foi de, respectivamente, 77,96±10,75 kg no grupo intervenção e 75,57±12,00 kg para o grupo controle. Notou-se que, tanto no início quanto no final da pesquisa, não houve diferença significante (p < 0,05) em relação às características físicas entre os dois grupos.

A Tabela 1 apresenta os resultados da medição dos diâmetros de panturrilha e tornozelo no início e no final da gestação. Verificou-se que as gestantes do grupo intervenção apresentaram menor aumento tanto no diâmetro da panturrilha quanto do tornozelo em relação ao grupo controle.

**Tabela 1:** Resultados das medições dos diâmetros (cm) de panturrilha e tornozelo das gestantes, no início e no final da gravidez. Botucatu, SP, 2017.

**Grupo**	**Local**	**Perna**	**Exame**	**Média**	**Mediana**	**Desvio padrão**	**Mínimo**	**Máximo**	**p-valor**
Intervenção	Panturrilha	Direita	Inicial	37,40	37,25	3,15	30,00	43,00	0,0174
			Final	37,70	37,50	3,37	29,50	43,50	
		Esquerda	Inicial	37,62	37,75	3,41	31,00	44,00	0,0174
			Final	37,92	38,00	3,51	30,00	44,00	
	Tornozelo	Direita	Inicial	22,97	23,00	1,85	19,00	26,00	0,1635
			Final	23,12	23,00	2,10	18,00	26,00	
		Esquerda	Inicial	23,08	23,00	1,90	19,00	26,00	0,1527
			Final	23,23	23,00	2,10	18,50	27,00	
Controle	Panturrilha	Direita	Inicial	35,62	35,00	3,73	30,00	44,00	< 0,0001
			Final	37,72	37,00	3,82	32,00	45,00	
		Esquerda	Inicial	35,69	35,00	3,86	30,00	45,00	< 0,0001
			Final	38,02	37,00	4,10	32,50	46,00	
	Tornozelo	Direita	Inicial	21,75	22,00	1,96	18,00	25,00	< 0,0001
			Final	23,62	24,00	2,21	19,50	28,50	
		Esquerda	Inicial	21,92	22,00	1,91	18,50	25,00	< 0,0001
			Final	23,90	24,00	2,17	20,00	28,50	

As diferenças médias, no início e no final da gestação, nos diâmetros de panturrilha direita, panturrilha esquerda, tornozelo direito e tornozelo esquerdo foram de, respectivamente, 0,30 cm, 0,30 cm, 0,15 cm e 0,15 cm no grupo intervenção e 1,95 cm, 1,95 cm, 1,73 cm e 1,87 cm no grupo controle (Tabela 2).

**Tabela 2:** Diferenças médias entre as medições dos diâmetros (cm) de panturrilha e tornozelo das gestantes, no início e no final da gravidez. Botucatu, SP, 2017.

**Local**	**Perna**	**Grupo**				**p-valor**
		**Intervenção**		**Controle**		
		**Média**	**Desvio padrão**	**Média**	**Desvio padrão**	
Panturrilha	Direita	0,30	0,65	1,95	0,99	< 0,0001
	Esquerda	0,30	0,65	1,95	1,14	< 0,0001
Tornozelo	Direita	0,15	0,57	1,73	0,73	< 0,0001
	Esquerda	0,15	0,56	1,87	0,96	< 0,0001

A Tabela 3 apresenta os dados referentes à percepção das gestantes sobre o uso das meias de compressão. A maioria das gestantes afirmou que não teve dificuldade para utilizar ou calçar as meias de compressão. Todas as gestantes relataram que sentiram diferença nos sintomas das pernas e que usariam as meias novamente.

**Tabela 3:** Distribuição das gestantes segundo a percepção sobre o uso das meias de compressão. Botucatu, SP, 2017.

**Questões sobre o uso das meias de compressão**		**Sim**		**Não**	**Total**	
	**n**	**%**	**n**	**%**	**n**	**%**
Teve alguma dificuldade quanto ao uso das meias de compressão?	11	36,67	19	63,33	30	100,00
Precisou de ajuda para calçar as meias de compressão?	6	20,00	24	80,00	30	100,00
Sentiu diferença nos sintomas das pernas com o uso das meias de compressão?	30	100,00	0	0,00	30	100,00
Durante o período de uso, em algum momento precisou retirar as meias de compressão?	11	36,67	19	63,33	30	100,00
Usaria as meias de compressão novamente?	30	100,00	0	0,00	30	100,00

## DISCUSSÃO

Neste estudo sobre o uso de meias de compressão em gestantes, verificou-se que o aumento nos diâmetros da panturrilha e do tornozelo foi menor nas gestantes que utilizaram meias de compressão, em comparação com o grupo controle, e que a medida apresentou boa aceitação entre as pacientes.

As veias varicosas, caracterizadas como veias subcutâneas que apresentam dilatação, tortuosidades e/ou alongamentos visíveis ou palpáveis clinicamente com o paciente em posição ortostática^14^^,^ 15, podem provocar manifestações clínicas indesejáveis, incluindo edema, dor, congestão, irritação da pele, câimbras musculares, peso, tensão e sensação de inchaço nos membros inferiores^15^. As intervenções não farmacológicas para tratamento de edema de perna e varizes incluem uso de meias elásticas de compressão, elevação da perna, qualquer forma de descanso, exercícios, reflexologia, imersão em água, fisioterapia e massagem^3^^,^ 16.

O edema de membros inferiores durante a gravidez é muito comum, atingindo cerca de 80% de todas as gestantes, e ocorre principalmente durante o terceiro trimestre gestacional, podendo ser caracterizado como edema fisiológico^17^^,^ 18. Diferentes técnicas podem ser utilizadas para avaliação dos níveis de edema dos membros inferiores, incluindo a medição da circunferência da perna, que pode ser realizada por meio de uma fita métrica ou com uma forma aprimorada de fita métrica, o Leg-O-Meter^18^. Outras abordagens, como reopletismografia, pletismografia por extensômetro e pletismografia a ar, podem ser usadas para avaliar as alterações associadas ao edema dos membros inferiores^18^. Outros métodos para a mensuração do edema de membros inferiores incluem avaliações optoeletrônicas, tomografia computadorizada, ressonância magnética e absorciometria de raios x de dupla energia. Entretanto, esses métodos são caros e de mais difícil acesso à população, em comparação ao método empregado no presente estudo^18^. Em uma recente pesquisa conduzida por Saliba et al.^9^, verificou-se que 70% das gestantes que não fizeram uso das meias queixaram-se de edema. Corroborando os achados do presente estudo, os autores também observaram que, no final da gestação, houve diminuição na prevalência de dor, edema e sensação de inchaço nos membros inferiores no grupo de gestantes que fizeram uso das meias de compressão, demonstrando a eficácia da terapia^9^.

É possível aventar que o efeito na prevenção do aumento do diâmetro da panturrilha e do tornozelo poderia até ser maior, pois não há como garantir que não houve uso incorreto das meias de compressão, embora ações tenham sido feitas para o controle do tratamento. O monitoramento do uso das meias de compressão feito por meio de telefonemas quinzenais periódicos foi extremamente importante para a realização da pesquisa, pois houve necessidade de entrega de novas meias, em decorrência de danos causados pelo uso, e até mesmo mudanças de dias e horários de agendamento para os exames.

A percepção sobre o uso das meias de compressão pelo grupo intervenção permitiu verificar a facilidade do emprego dessa medida profilática e terapêutica. Todas as gestantes relataram que sentiram diferença nos sintomas das pernas e que usariam novamente as meias de compressão. Os achados desta pesquisa estão de acordo com o estudo sobre aceitação de meias de compressão realizado por Allegra et al.^19^, que verificaram redução no sintoma de dor nas pernas em gestantes que utilizaram meias de compressão. Os autores também observaram que a melhora nos sintomas esteve associada à regularidade na utilização das meias, demonstrando a importância do seu uso contínuo para a melhoria da qualidade de vida das gestantes^19^.

Questões importantes relacionadas à eficácia das meias são: a compressão necessária para atingir os efeitos desejados e qual o efeito da compressão sobre o volume dos membros. Resultados do uso de meias de compressão, com prescrição de 20-30 mmHg de compressão durante 8 h por dia desde a 12ª semana de gestação, demonstraram refluxo na VSM e na veia safena parva em 0/30 pacientes no grupo tratado e em 16/30 no grupo que não fez uso das meias, analisadas por meio de exame eco-Doppler no final da gestação^20^. Thaler et al.^21^ utilizaram meias com diferentes graus de compressão e verificaram refluxo na junção safeno-femoral em 1/27 pacientes do grupo tratado e em 4/15 do grupo controle. Em estudo realizado com tomografia computadorizada para verificar o efeito *in vivo* promovido pelas meias elásticas em veias superficiais na posição deitada, observou-se que uma compressão de 36 mmHg reduziu o calibre da VSM em 70%^22^. Esses achados evidenciam a importância da escolha adequada do protocolo de prescrição do uso das meias de compressão para obtenção dos resultados almejados.

Reconhecendo a TVP e a embolia pulmonar como algumas das principais causas de morbidade e mortalidade na gravidez, um estudo de coorte foi realizado com gestantes atendidas em um Programa de Atenção à Saúde da Gestante na clínica obstétrica de um hospital de Cremona, Itália, para avaliar os efeitos do uso de meias de compressão de 12-18 mmHg acima do joelho, com e sem administração associada de heparina de baixo peso molecular, na prevenção dessas complicações. Os autores concluíram que ambas as intervenções se mostraram seguras e úteis para a redução da incidência de tromboembolismo venoso entre as gestantes^23^. Ademais, evidências sugerem que a utilização de meias de compressão de 20-30 mmHg pode ser adotada como medida preventiva para minimizar o risco de tromboembolismo venoso em gestantes durante viagens de longa distância^24^.

Nesta pesquisa, todas as gestantes fizeram uso diário das meias por pelo menos 8 h. Houve monitoramento, por meio de contato telefônico, e a abordagem da questão deu-se em termos de períodos do dia (manhã, tarde e noite). Não foi solicitado o registro exato do tempo de utilização diária das meias de compressão, pois a imposição de muitas condições poderia diminuir a adesão à participação. Salienta-se que, mesmo que cerca de 1/3 das gestantes tenha apresentado alguma dificuldade no uso das meias de compressão e tenha precisado retirá-las em algum momento do período do estudo, não houve longas interrupções no uso das meias, pois se tratou de situações pontuais referentes a eventos relacionados à necessidade de substituição das meias devido a danos causados pelo uso constante. Nesses casos, seguindo as orientações recebidas, as gestantes entraram em contato com os pesquisadores para recebimento imediato de novas meias, evidenciando a importância da continuidade e da aderência ao tratamento para atingir os resultados desejados^20^. O fato de as gestantes terem sido acompanhadas, por meio de contato telefônico, talvez tenha sido um diferencial positivo para a aderência e o uso recomendado das meias de compressão, o que, de certa forma, possibilitou a identificação das dificuldades apontadas. Estudo de Adamczyk et al.^25^ demonstrou que a terapia de compressão foi bem tolerada por mulheres grávidas e que a melhora nos sintomas foi percebida com o uso diário por pelo menos 4 h.

Destaca-se que, no presente estudo, foram incluídas tanto gestantes primigestas quanto mulheres que tiveram uma ou mais gestações, de modo que os grupos se apresentaram equilibrados quanto a essa variável. Esse foi um diferencial desta pesquisa, pois, na literatura científica, a maior parte dos estudos incluem somente gestantes primigestas. Estudos demonstram que as mulheres multíparas apresentam um risco maior de desenvolver veias varicosas ao longo do tempo, independentemente do ganho de peso associado à gravidez^11^^,^ 26. Essas alterações estão presentes em aproximadamente 13% das mulheres primíparas, 30% das secundíparas, e até 57% das multíparas^26^. Ademais, evidências sugerem que a multiparidade (≥ 4 partos) representa um fator de risco relevante para a ocorrência de tromboembolismo venoso durante a gravidez^12^.

Conclui-se que as meias de compressão foram uma medida eficaz na prevenção de edema em membros inferiores de gestantes, as quais apresentaram percepção positiva quanto à sua utilização.
